# Supplementary material for: Measuring Growth, Resistance, and Recovery after Artemisinin Treatment of Plasmodium falciparum in a single semi-high-throughput Assay
Source: bioRxiv. 2024 Nov 11:2024.11.11.623064. Preprint. [Version 1] doi: 10.1101/2024.11.11.623064 (PMC11601240; doi:10.1101/2024.11.11.623064)
Supplement: Supplement 1 [file NIHPP2024.11.11.623064v1-supplement-1.pdf]

bioRxiv preprint doi: https://doi.org/10.1101/2024.11.11.623064; this version posted November 11, 2024. The copyright holder for this preprint (which was not certified by peer review) is the author/funder, who has granted bioRxiv a license to display the preprint in perpetuity. It is made available under aCC-BY 4.0 International license.

Additional files

Additional file 1. Table of primers and reagents used for quantitative PCR.

Volume	Reagent	Supplier
5 µL	2X Phusion Blood Direct Mix Buffer	ThermoFisher, cat # F547L
0.25 µL	pfCRT Forward Primer<br>(AGATTTCGTAACTTTGGTAAGTGTG)	Integrated DNA Technologies
0.25 µL	pfCRT Reverse Primer<br>(ATGAACGAACAAGCCATTTGAT)	Integrated DNA Technologies
1.4 µL	7.5X SYBR Green in H_2O	Invitrogen, cat # S7585
0.1 µL	Phusion enzyme	ThermoFisher, cat # F547L
3 µL	Thawed culture sample	-

Additional file 2. Table of cycling conditions used for quantitative PCR

Cycle Step	Temp.	Time	Cycle No.
Initial Denaturation	95.0°C	20 s	1
Denaturation<br>Annealing<br>Extension	95.0°C<br>62.3°C<br>65.0°C	1 s<br>30 s<br>15 s	30

bioRxiv preprint doi: https://doi.org/10.1101/2024.11.11.623064; this version posted November 11, 2024. The copyright holder for this preprint (which was not certified by peer review) is the author/funder, who has granted bioRxiv a license to display the preprint in perpetuity. It is made available under aCC-BY 4.0 International license.

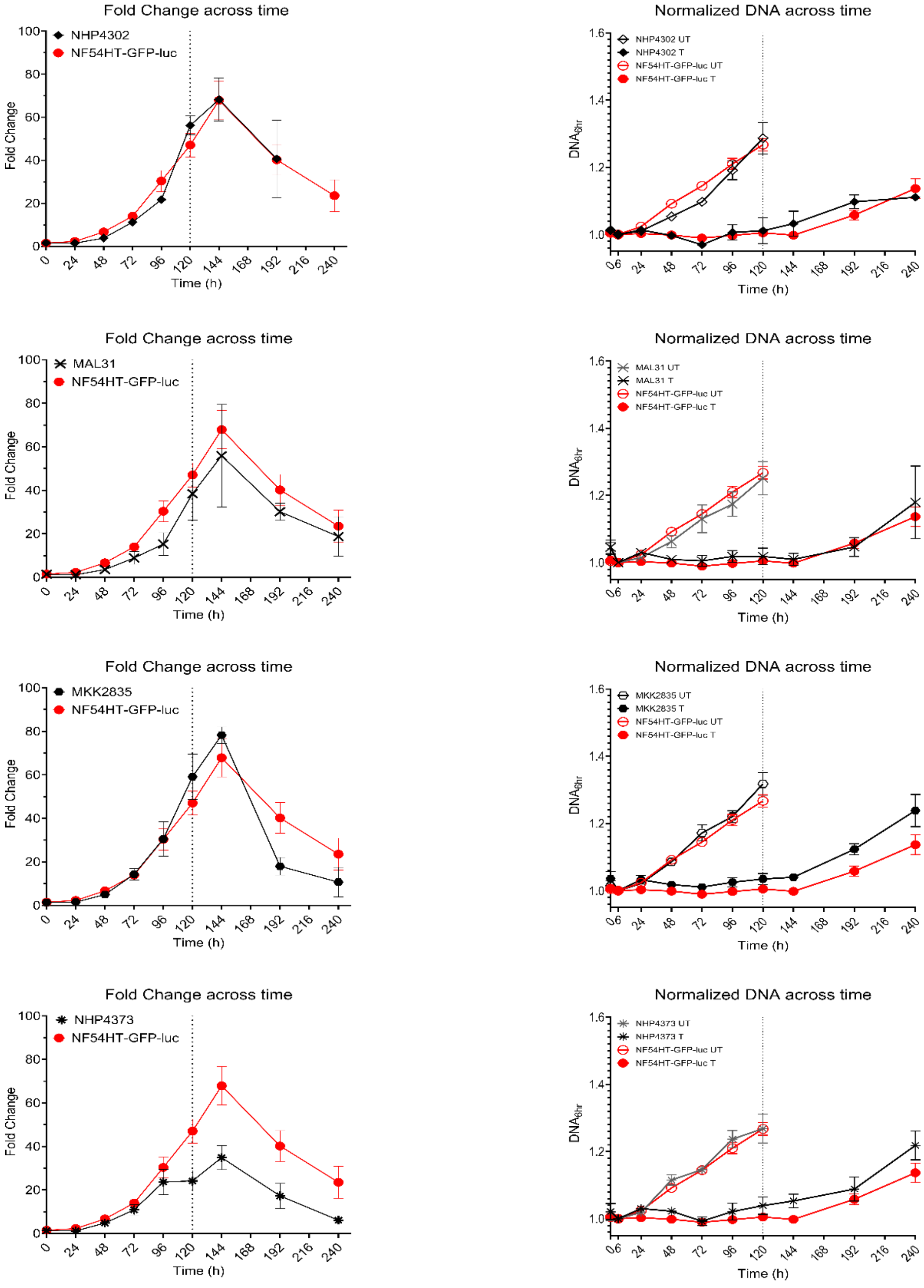

bioRxiv preprint doi: https://doi.org/10.1101/2024.11.11.623064; this version posted November 11, 2024. The copyright holder for this preprint (which was not certified by peer review) is the author/funder, who has granted bioRxiv a license to display the preprint in perpetuity. It is made available under aCC-BY 4.0 International license.

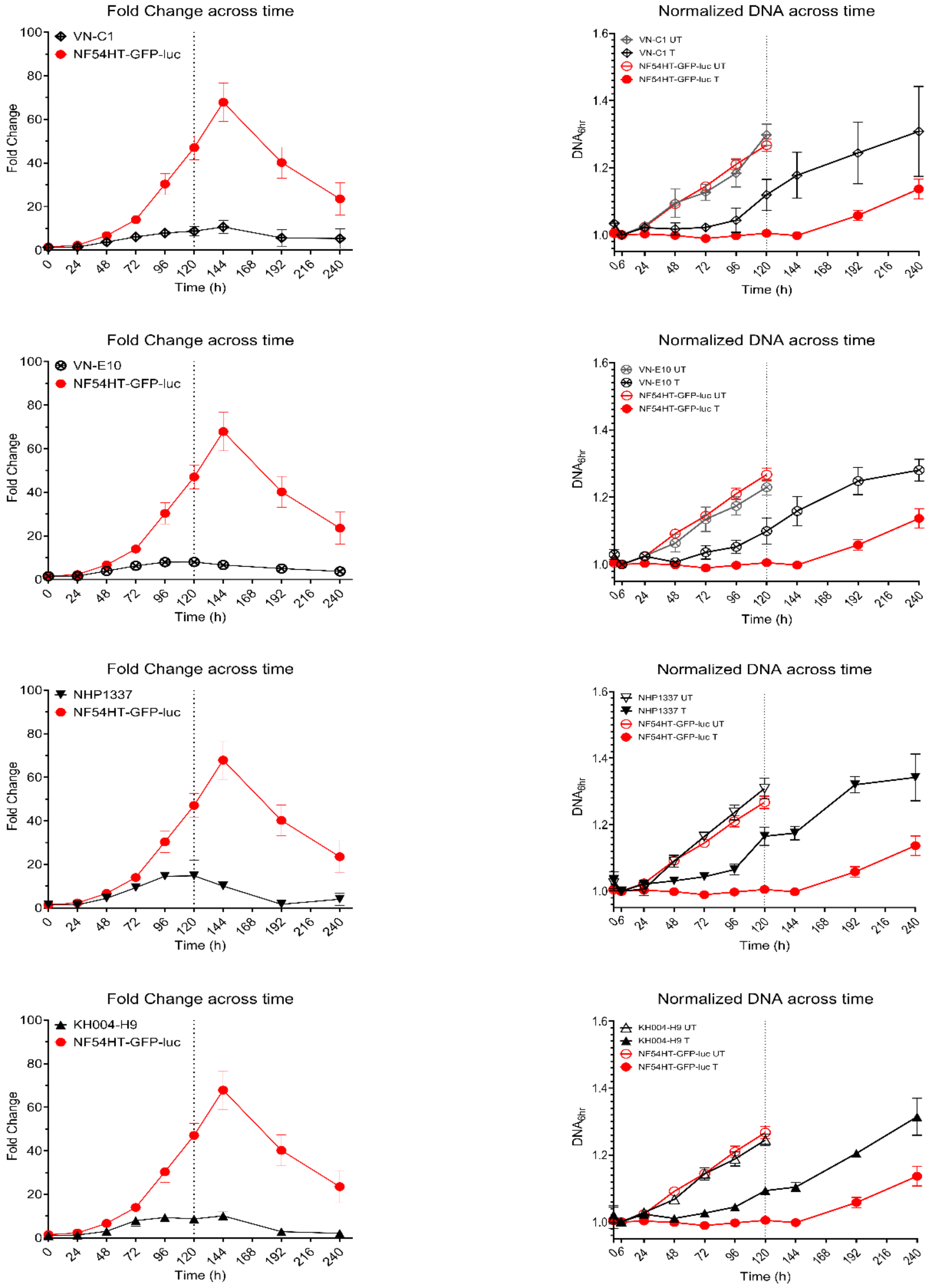

Additional file 2. Fold Change and DNA content of treated and untreated cultures across 10-day time series for additional clinical isolates not shown in main figures. Each isolate is shown with NF54HT-GFP-luc for reference. Data is reported as mean ± SEM of at least three biological replicates.

bioRxiv preprint doi: https://doi.org/10.1101/2024.11.11.623064; this version posted November 11, 2024. The copyright holder for this preprint (which was not certified by peer review) is the author/funder, who has granted bioRxiv a license to display the preprint in perpetuity. It is made available under aCC-BY 4.0 International license.

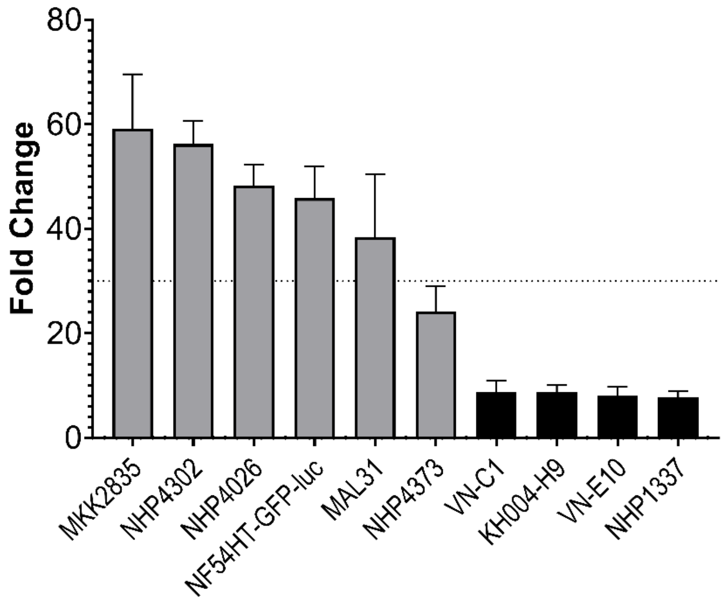

Additional file 3. Distribution of Resistance phenotypes for clinical isolates and CRISPR/Cas9 edited K13 mutant parasite lines. Parasites are ordered by increasing level of resistance and include parents of recent genetic crosses (NF54HT-GFP-luc × NHP4026, MKK2835 × NHP1337, MAL31 × KH004-H9), NHP4302 and additional isolates (NHP4373, VN-C1, VN-E10). Parasites with a K13^{C580Y} mutation are marked in black and K13 wildtype parasites are marked in grey. The dashed line at 30-Fold Change represents the threshold for resistance, as previously reported (27). Data is reported as mean ± SEM of at least three biological replicates.

bioRxiv preprint doi: https://doi.org/10.1101/2024.11.11.623064; this version posted November 11, 2024. The copyright holder for this preprint (which was not certified by peer review) is the author/funder, who has granted bioRxiv a license to display the preprint in perpetuity. It is made available under aCC-BY 4.0 International license.

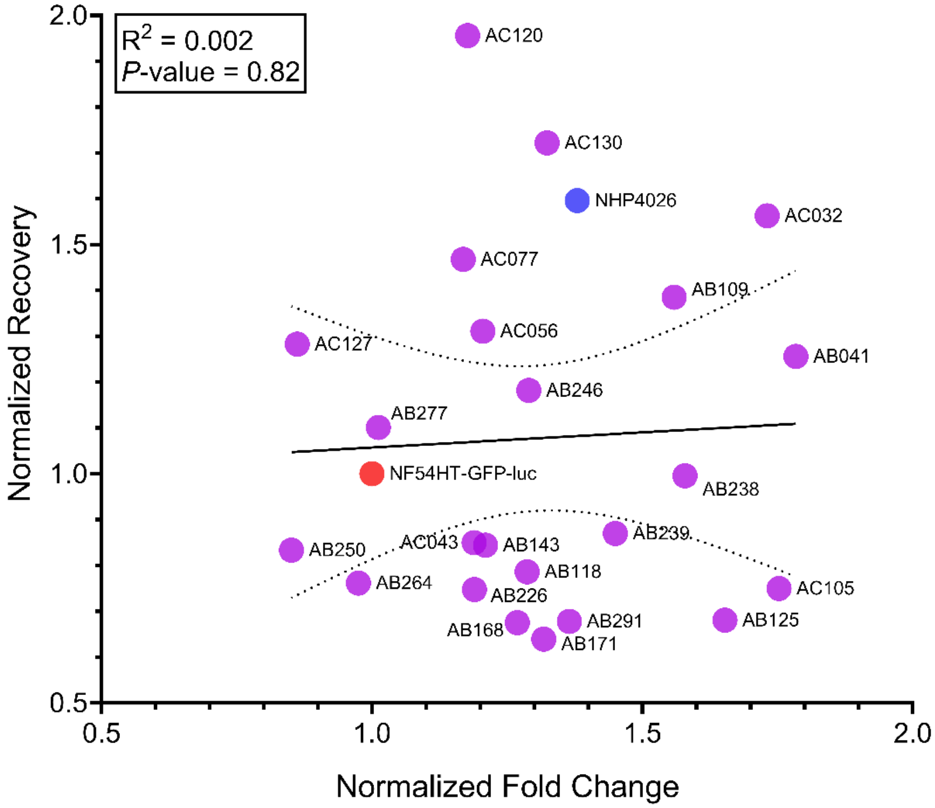

Additional file 4. Scatterplot of Resistance vs Recovery phenotypes measured in 23 progeny and parents of the NF54(HT-GFP-luc) × NHP4026 genetic cross. Linear regression does not show a significant association Resistance and Recovery (R^2 = 0.0021, F(1, 23) = 0.0501, *P*-value= 0.8249). The dotted lines denote the 95% confidence interval around the best fit line ($y = 0.06616x + 0.9912$). Linear regression was performed using mean phenotypes of at least three biological replicates and phenotype values are normalized to the NF54HT-GFP-luc parent.

bioRxiv preprint doi: https://doi.org/10.1101/2024.11.11.623064; this version posted November 11, 2024. The copyright holder for this preprint (which was not certified by peer review) is the author/funder, who has granted bioRxiv a license to display the preprint in perpetuity. It is made available under aCC-BY 4.0 International license.

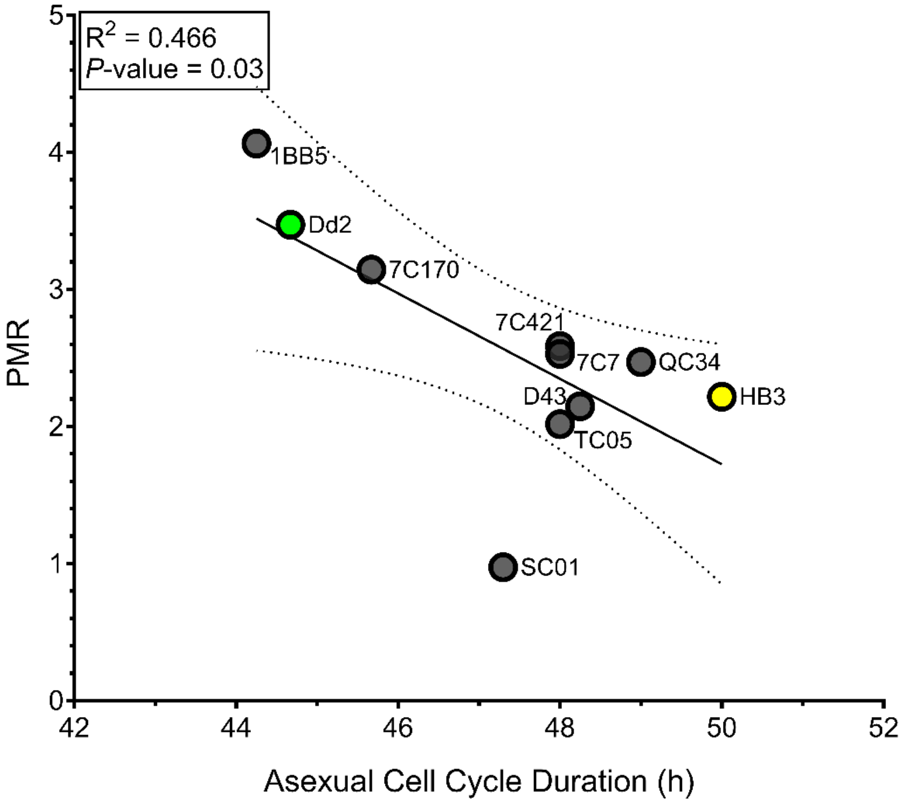

Additional file 5. Scatterplot of PMR and asexual cell cycle duration measured in eight progeny and parents of the HB3 × Dd2 genetic cross. Linear regression between phenotypes shows a significant linear association that PMR increases as cell cycle duration decreases and 46.6% of variation is shared between the phenotypes (R^2 = 0.4661, F(1, 8) = 6.984, *P*-value = 0.0296). The dotted lines denote the 95% confidence interval around the best fit line ($y = -0.3115x + 17.3$). Linear regression was performed using mean phenotypes of at least three biological replicates or accessed from previously published sources (31).

bioRxiv preprint doi: https://doi.org/10.1101/2024.11.11.623064; this version posted November 11, 2024. The copyright holder for this preprint (which was not certified by peer review) is the author/funder, who has granted bioRxiv a license to display the preprint in perpetuity. It is made available under aCC-BY 4.0 International license.

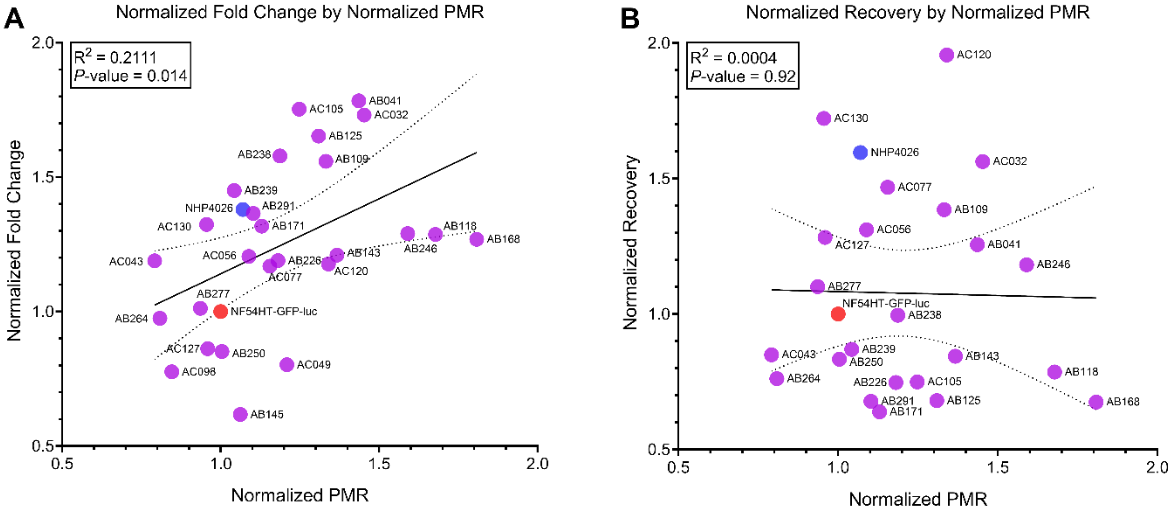

Additional file 6. Scatterplot of PMR vs Resistance or Recovery phenotypes in progeny and parents of the NF54HT-GFP-luc × NHP4026 genetic cross. A) Linear regression of PMR vs Fold Change shows a significant and positive linear association with 21.1% of variation shared between the phenotypes (R^2 = 0.2111, F(1, 26) = 6.956, *P*-value= 0.0139). The dotted lines denote the 95% confidence interval around the best fit line ($y = 0.5582x + 0.5824$). **B)** Linear regression of PMR vs Recovery shows no significant association (R^2 = 0.0004, F(1, 23) = 0.009, *P*-value= 0.9234). The dotted lines denote the 95% confidence interval around the best fit line ($y = -0.02936x + 1.113$). Linear regression was performed using mean phenotypes of at least three biological replicates and phenotype values are normalized to the NF54HT-GFP-luc parent.
